# Progress in Infrared Photodetectors Since 2000

**DOI:** 10.3390/s130405054

**Published:** 2013-04-16

**Authors:** Chandler Downs, Thomas E. Vandervelde

**Affiliations:** Renewable Energy and Applied Photonics Laboratories, Electrical and Computer Engineering Department, Tufts University, 161 College Ave., Medford, MA 02115, USA; E-Mail: chandler.downs@tufts.edu

**Keywords:** infrared photodetectors, IRPDs, bulk detectors, QWIPs, QDIPs, DWELLs, superlattices

## Abstract

The first decade of the 21st-century has seen a rapid development in infrared photodetector technology. At the end of the last millennium there were two dominant IR systems, InSb- and HgCdTe-based detectors, which were well developed and available in commercial systems. While these two systems saw improvements over the last twelve years, their change has not nearly been as marked as that of the quantum-based detectors (*i.e.*, QWIPs, QDIPs, DWELL-IPs, and SLS-based photodetectors). In this paper, we review the progress made in all of these systems over the last decade plus, compare the relative merits of the systems as they stand now, and discuss where some of the leading research groups in these fields are going to take these technologies in the years to come.

## 1. Introduction

Infrared photodetectors (IRPDs) are a technology with wide-ranging and rapidly expanding applications in the modern world. Ever since Friedrick William Herschel discovered the presence of infrared radiation in sunlight in the early 19th century, people have tried various means to detect and analyze this spectrum of light invisible to the naked eye [1]. The earliest practical IR detectors were developed by Macedonio Melloni in the mid-19th century [2]. These detectors were thermopiles that functioned by thermal conduction, typically by relying on the differences in thermal expansion of two dissimilar metals. In 1917, Case developed what could be considered the first modern photodetector, when in his search for materials which exhibited variable resistances depending on whether light was shined on them [3]. In this research he noted a number of materials, such as lead sulfide, exhibited responses out into the IR regime. These were the first IR detectors to operate using quantum effects rather than conductive ones, and it was this technology that fathered the field of IRPDs as we know them today [4].

Applications currently utilizing IRPDs span military (e.g., navigation, night vision, weapons detection), commercial (e.g., communications, aerospace, medical imaging), public (e.g., atmospheric sounding, pollution control, meteorology, environmental monitoring), and academic (e.g., astronomy) domains-with new uses constantly arising as the various IRPD technologies become more established [5–11]. As such, researchers have invested tremendous time and resources into developing and improving various IRPD technologies to further serve these applications. Of particular note are the advances since the new millennium. Within the past twelve years, established technologies have grown into commercial successes, nascent technologies have grown into thriving hubs of research, and new technologies have been discovered and begun to be investigated.

The world around us is a large source of infrared radiation and IRPDs can be useful in a wide array of applications utilizing it. This ubiquity is due to the fact that all objects will emit an IR spectrum based on their temperature. This emission spectrum can be approximated by wavelength λ as blackbody radiation, which can be characterized according to the blackbody's temperature *T* by Equation (1) [12]:

(1) $$e_{B}\;(\lambda ,T)\;d\lambda =\frac{2\pi hc^{2}d\lambda }{\lambda^{5}[e^{hc/\lambda kT}-1]}$$

This equation illustrates why IRPDs have received so much interest of late. It implies that an object at room temperature will emit IR radiation with a peak intensity of around 9.5 μm, with detectable IR emissions for wavelengths microns away from this center value. While not every portion of the IR spectrum is ordinarily present due to natural blackbody radiation (a blackbody would need to be at a temperature of over 4,000 K to have peak light emissions at the edge of the visible spectrum), these sources do emit across large portions of the IR spectrum.

**Figure 1.**
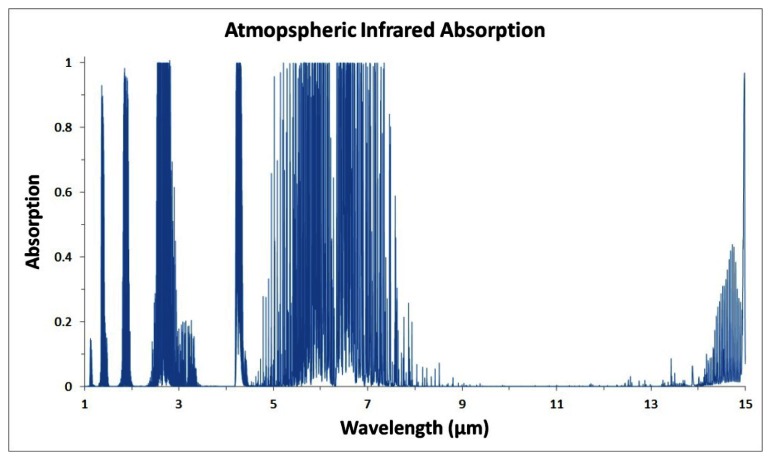
A plot of the atmosphere's absorption spectrum [16]. Note that light emitted in the 2.5–3.5 and 5–7 μm ranges are rapidly absorbed by the atmosphere.

One complication for IRPDs is atmospheric absorption. As IR radiation propagates through the atmosphere, some wavelengths are readily absorbed due to the molecules present in the air. Of greatest concern to IR radiation are the absorption spectra of water vapor and various hydrocarbons [13], whose absorption spectra cut large infrared windows of the atmospheric transmission spectrum. These infrared windows (seen in Figure 1) do not necessary preclude the use of IRPDs at these wavelengths, but they can significantly interfere with device performance at those wavelengths [14,15].

The wavelengths of light associated with IRPDs are those longer than visible light (longer than 700 nm). This is a truly massive range, one no single detector technology is able to completely span. Comparisons between the performance of devices at vastly different absorption wavelengths can be difficult to make, given that performance metrics may vary by orders of magnitude across the IR spectrum. In hopes of encouraging reasonable comparisons between devices, the IR spectrum has been divided (as best as is possible) into a number of sub-regions. Where possible, these divisions have been made at convenient or recognizable points to aid in distinguishing between the regions.

Closest to the visible spectrum is the near infrared (NIR) regime, ranging from 700 nm to about 1.0 μm, corresponding to the cutoff for absorption by silicon. From the 1.0 μm cutoff to 3 μm is the short-wave infrared (SWIR) regime, with cutoff defined by one of the atmospheric windows. This is followed by the mid-wave infrared (MWIR) regime, ranging from 3 μm to 5 μm, again with the cutoff defined by the atmospheric window. The long-wave infrared (LWIR) regime ranges from 8 μm to 12 μm, while the very long-wave infrared regime (VLWIR) amasses everything beyond 12 μm [5]. There is a range of wavelengths, from 5 μm to 8 μm, which does not fall into any of these categories. This range of wavelengths corresponds to a large region of atmospheric IR absorption. This absorption does not preclude development of IRPDs at these wavelengths provided they are detecting nearby sources of radiation in that range, but it can make the transmission of those wavelengths more difficult, decreasing the effective range of the detectors [5]. In this paper, we will attempt to constrain our device comparisons to within the same wavelength regime, even within the same technology, so as to avoid any improper comparisons.

Over the years, a large number of metrics have been developed to measure the performance of IRPDs. In this review, we will focus on the most commonly reported device metrics. Peak absorption wavelength will be used to separate detectors into the different regimes listed above. For single pixel detectors, the most common metrics are dark current density, peak detectivity, peak responsivity, and operating temperature. Lower dark current densities allow lower response signals to be detected, a desired trait in all devices. Comparisons of dark currents at a given temperature within a single technology are a valid means of determining superior performance, but comparisons between IRPD technologies must be taken with a grain of salt. Different technologies (or even the same technology operating in a different wavelength regime) may produce dramatically different signal current densities operating at similar conditions. The amount of signal current generated for a given input power of IR radiation (measured for a specific wavelength) is the responsivity of the device and is defined in Equation (2):

(2) $$R=\frac{e\eta g}{hv}$$

where *η* is the quantum efficiency (the percentage of generated carriers that are extracted from the device) of the detector and *g* is the photoconductive gain (the number of carriers that are generated by the device structure and applied bias for every carrier generated by an absorbed photon) [17]. As with dark current density, comparisons of responsivity are valid within specific technologies under comparable conditions, but are deceptive outside of those limitations. In general, higher responsivities will result in better device performance. For the purposes of this paper, comparisons concerning responsivity will be done according to peak responsivity values reported independent of wavelength. Comparisons of responsivity values will also be limited to devices of the same technology under similar operating conditions.

Another favored metric used to delineate IRPD performance is specific detectivity (D*). Specific detectivity incorporates aspects of both the dark current density and responsivity of a device to provide a comparison of the amount of signal current generation for a given amount of noise at a specific wavelength, defined in Equation (3):

(3) $$D^{*}=\frac{R_{p}\sqrt{A\Delta f}}{i_{n}}$$

where *R_p_* is the peak responsivity, *A* is the cross-sectional area of the IRPD, *Δf* is the bandwidth of the device, and *i_n_* is the noise current [17]. High specific detectivities indicate a larger signal current generated for a given amount noise, which allows for better signal detection. Further, comparisons of specific detectivities across different technologies and operating conditions are reasonable, making this one of the most useful analytic metrics. Closely related to the three previous metrics is the maximum operating temperature of the device. The higher the temperature an IRPD is operated at, the more noise will be present. This in turn increases dark current and reduces specific detectivity. Once the size of the dark current is large enough to make the signal current indistinguishable, the device will no longer function. Photodetectors with higher maximum operating temperatures require less cooling to maintain functionality. The need for less cooling can dramatically reduce cost of operation for these devices and allow the devices to be used in environments and situations where liquid cryogens are not available.

Focal plane arrays (FPAs) necessitate a handful of additional performance metrics in addition to those used to describe single pixel detectors in order to address overall image quality. The most obvious is simply the number of pixels present in the FPA. A larger pixel count enables more precise imaging, generally a desirable feature. Similarly, pixel size is important, with smaller pixels allowing for more precise imaging. However, since most FPAs are fabricated using standard read out integrated circuit (ROIC) packaging, there is more a series of benchmarks to be met in terms of pixel size and pixel count. A more useful metric for FPAs is the noise equivalent temperature difference (NETD), which can be used to approximate the minimum resolvable temperature difference of the FPA, defined in Equation (4):

(4) $$\text{NETD}=\;\frac{i_{n}}{R}\frac{dP}{dT_{B}}=\frac{i_{n}hvP}{k_{B}T_{B}^{2}}$$

where *P* is the incident power of light on the detector, *T_B_* is the blackbody radiation temperature, and *k_B_* is the Boltzmann constant [18]. A number of device parameters, from pixel size to specific detectivity, are taken into account in this parameter. A smaller NETD is preferable, indicating a capability for finer, more precise imaging. We will additionally make note of any multicolor FPAs which incorporate multiple different wavelengths of IR detection, particularly if the wavelengths of detection are significantly different. Such multispectral imaging has many advantages for a multitude of applications.

In this review, the progress made in each of the most researched IRPD technologies since 2000 will be examined in its own section. Each section will begin with a brief description of the technology in question and highest performing devices in 2000. This will be followed with a chronicle of the major trends in the technology over the ensuing twelve years, e.g., popular material choices or inclusion of specific structures in devices. A current state of the technology will also be provided for each section. If applicable, closely related technologies will be examined in a subsection.

## 2. Bulk Detectors

As of 2000, bulk detectors were the most common IRPD technology. A wide variety of materials were utilized in various applications, but most popular were HgCdTe (MCT) and InSb. The active portions of these detectors are generally photodiodes, though there is great variety in the structure and fabrication of the specific photodiodes. There are numerous fabrication methods, each having various strengths and weakness based on the specific IRPD design (e.g., front- or back-illuminated, epitaxial or implanted layers, operation in the photoconductive or photovoltaic regime, *etc.*), but these are beyond the scope of this review [7,19]. Bulk detectors were uniformly the most popular detectors at the outset of the 21st century for a number of reasons, largely due to the relative maturity of the technologies. Bulk detectors, because of their relative ease of fabrication, were first developed significantly earlier than other technologies.

### 2.1. HgCdTe (MCT)

Since the development of MCT in 1959 and the creation of the first detector using it in 1962, MCT has been one of the most popular materials for IRPDs [20–22]. By 2000, the technology was fairly mature. Single pixel and focal plane array (FPA) detectors had been extensively studied in laboratory settings, with the research from those devices being used as the groundwork for a number of commercial ventures. As such, published research in this area was actually not as widespread as in other IRPD technologies over this timeframe. Much of the research performed was focused less on direct improvement in performance of devices, but instead on addressing specific difficulties encountered by the technology.

**Figure 2.**
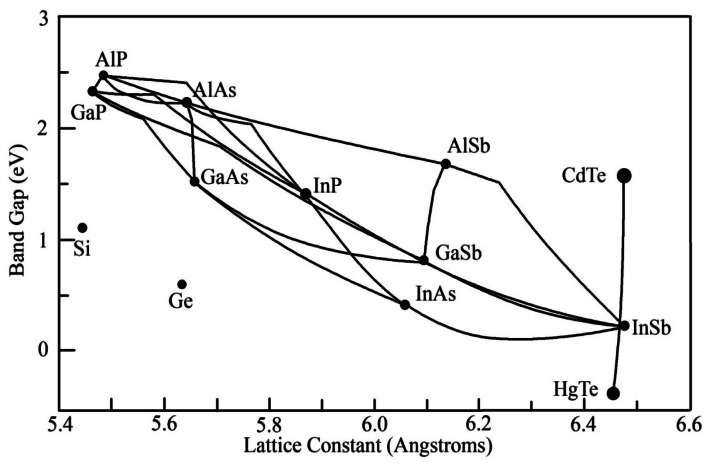
A lattice constant *vs.* band gap plot of common semiconductor materials.

The scope of this research becomes much clearer when examining the strengths and weaknesses of the MCT technology as a whole. Some of its greatest strengths have been touched on already, namely the established history of the technology and the relative simplicity of fabrication compared to a number of the later technologies. There are a number of methods utilized to grow MCT devices, including metalorganic vapor phase epitaxy (MOCVD), liquid phase epitaxy (LPE), and molecular beam epitaxy (MBE), which allows for more flexibility in fabrication [19–25]. MCT detectors tend to have very high responsivities and competitive detectivities when compared with quantum structures utilized in other technologies [26]. As MCT is a ternary material, by varying the composition of the material it is possible to span all of the wavelengths between the constituent binary materials, as can be seen in Figure 2. With MCT, this is a large range, spanning all wavelengths between 0.7 μm and 25 μm and three atmospheric windows [19,23,27–29]. However, despite the wide range of wavelengths MCT spans, there is very little variation in the value of the lattice constant across that range [23]. This allows for simplification of many of the enabling techniques used in IRPDs (substrate selection, ROIC usage, *etc.*) to be used across all MCT compositions. MCT devices also tend to have very short integration times and very low junction capacitances compared to other bulk detectors due their low dielectric constant, both of which enhance its capability to detect fast moving objects [4,19,30].

With these advantages come a number of disadvantages. The most noteworthy of these is that dark currents in MCT devices tend to be extremely large, much larger than in other devices, influenced heavily by band-to-band tunneling [31]. MCT devices also have growth related issues due to differences in the densities and melting points of the constituent materials, the relative softness of the final material, and a number of crystalline properties of the material (e.g., high interdiffusion coefficients, low bond strength, and a high rate of dislocation formation) [4,15,28]. These issues can make precision growths of MCT extremely difficult, which lead to a number of other device issues. One of the major issues, particularly as the band gap of the material decreases, is finely controlling the composition of the material [28,32]. As the band gap goes down towards the LWIR and VLWIR regimes, variation of the mole composition of MCT by 0.001 can have drastic effects on the performance of a detector. That level of control is extremely difficult, regardless of the growth method. Additionally, it is hard to achieve the kind of uniformity across a wafer that is necessary for large area FPAs, which has led some to question MCTs suitability for some applications [15,26,32,33]. This problem is only exacerbated when the issue of multicolor FPAs is considered [30].

As mentioned previously, by 2000 MCT IRPDs were already an established and mature technology. Much of the research performed since then has focused on addressing some of the issues mentioned above or expanding the commercial utility of the technology, particularly with FPAs. This is partially due to the increased research performed by commercial organizations, as opposed to government or academic organizations. Companies such as Raytheon, BAE, Sofradir, and many others have published numerous papers on their progress with commercial products. In the early part of the decade, these papers generally focused on small- to mid-sized FPAs, generally operating in the NIR, SWIR, MWIR regimes. The majority of these devices were operated around liquid nitrogen temperatures (77–100 K), though there were a couple of noteworthy exceptions [19,34–37]. A few standout devices operating in the NIR and SWIR regimes, where dark currents are minimized with the corresponding larger bandgaps, were capable of functioning at elevated temperatures, up to around 250 K [24,38,39]. The fabrication method most favored for these devices was LPE, but MBE growth was growing in popularity, particularly with devices requiring large cross-sectional areas due to MBE's finer growth control. As the decade progressed, commercial interests pushed towards larger FPAs (with the largest being a 2,560 × 512 pixel FPA using a double layer heterojunction design grown by MBE by Raytheon [40]) and longer detection wavelengths, pushing detection out to as far as 13 μm [41]. Published data also showed attempts to incorporate multicolor detection, starting with dual-color detection, into the technology with reasonably successful results [40,42]. Other trends showed an increased reliance on MBE growth for what should be considered prototype devices, with the likely intention to eventually shift to faster processes after the initial structures are completed [43–45].

Research performed in various academic and governmental laboratories has had a slightly different focus than commercial research. As early as 2000, MBE was regularly used in the growth of MCT detectors. This is not wholly unexpected, as MBE does allow for better control of material quality for the small numbers of cells that are generally grown in these settings. In the early half of the decade, the bulk of published research centered mainly on two subjects: LWIR detectors and growth of MCT on silicon substrates. LWIR detection was being researched as an extension of the progress made in SWIR and MWIR previously. These devices were usually small to mid-sized FPAs (e.g., 384 × 288) operated at liquid nitrogen temperatures and focused on using MBE growth to achieve the level of composition control necessary for precise LWIR regime absorption [28,46,47]. Research focused on silicon substrates looked to address cost concerns that could arise from typical MCT substrates (most commonly CdZnTe). Growth of MCT on silicon in the early part of the decade was performed exclusively by MBE, as the lattice mismatch between various MCT alloys and the silicon substrate can be as large as 19% [25,48–51]. Research was also performed to enable improved room temperature performance of MCT detectors. While as of 2000 room temperature operation was feasible, performance was lackluster, necessitating further research [52].

Moving through the later part of the decade, research on MCT detectors by universities and government agencies had less of a unified focus. Research on deposition of MCT onto Si substrates slowed to a near halt [53]. Research continued improving the performance of high operating temperature devices, both in terms of detectivity as well as extending the wavelengths at which this could be achieved. Attempts were made to improve the detectivity of devices (e.g., by using multiple absorbing layers) [23] as well as decrease the dark current density (e.g., including Auger suppression layers) [54]. Moreover, research continued extending the operational wavelengths for MCT devices, with functional detectors pushing into the VLWIR regime up to 14 μm [7] and D* of LWIR detectors reaching up to 1.9 × 10^11^ cm Hz^1/2^/W [55].

Despite these local areas of focus, there was also a great range of research on individualized topics [56,57]. Further research was performed on multicolor detectors, focusing on moving into the MWIR and LWIR regimes utilizing multiple absorbing layers [58]. Amorphous MCT was investigated as a detector material, as it is easier to grow on silicon. The performance of the detectors exhibited lower detectivities (on the order of 10^8^ cmHz^1/2^/W) than crystalline MCT, but did exhibit the possibility of near-room temperature operation [32]. Experiments were also performed using resonant cavity enhancements for improved absorption of incident light at specific wavelengths. This design does have some special considerations, as one of the characteristics of MCT detectors is the wide absorption bands, whereas the resonant cavity enhancements tend to have narrow absorption bands. These detectors have shown improved absorption at specific absorber wavelengths, but reduced absorption at other wavelengths [59].

### 2.2. InSb

InSb photodetectors have been fabricated commercially since the late 1950's. InSb photodetectors, like MCT photodetectors, are bulk detectors. Most detectors utilize some form of a p-n junction (including p-i-n, p^+^-p-n^+^, *etc.*), though many incorporate a metal-insulator-semiconductor (MIS) contact structure [4,60]. In the early part of the decade, InSb was the material of choice for NIR and some MWIR applications, in part because it was an established technology and in part because the material's band gap falls squarely in that range. Further, the material is often combined with various other column IV or VI materials, most commonly arsenic. Attempts have been made to utilize thallium or bismuth to achieve a narrower band gap, but including small amounts of these materials in InSb (<5%) led to great difficulty in material fabrication and dramatically limited device detectivity and responsivity [61,62]. InSb FPAs were capable of pixel counts roughly similar to MCT FPAs at the start of the decade [4].

InSb detectors have several notable strengths. Its band gap (0.17 eV (∼7.3 μm) at 300 K, 0.23 eV (∼5.4 μm) at 80 K), allows it to function comfortably as a binary material in the MWIR regime. Its utility in the MWIR is expanded when arsenic is included in a ternary material. InAsSb is capable of spanning all wavelengths between InSb and InAs (band gap of 0.35 eV (∼3.5 μm)). This flexibility allows InAsSb to be tuned to most wavelengths in the MIR regime. Further, because the difference in band gaps between InSb and InAs is much less than that between HgTe and CdTe, the tolerances in terms of material composition when designing specific operating wavelengths are eased [60]. This also limits the utility of these devices, as it can be very difficult for InSb or InAsSb devices to operate competitively outside the MWIR regime. InSb-based detectors also tend to have high device stability, lower fragility, and better device yield than MCT devices [60,63,64].

The use of InSb for photodetectors has also been associated with a number of fabrication difficulties. As can be seen in Figure 2, InAsSb spans a wide range of lattice constants. As such, while there are larger tolerances for material composition in terms of absorption wavelength, there are much stricter tolerances in terms of lattice matching to a substrate. At the beginning of the decade, the substrates for most of these devices were InSb, as the lattice mismatch between even InSb and GaSb was large enough to cause issues. This issue was exacerbated with more common substrates like GaAs or silicon. When attempts were made to utilize GaAs or Si substrates, the InSb/substrate interface suffered from high dislocation densities despite the use of a number of advanced techniques (e.g., inclusion of buffer layers) to ease the strain. The high dislocation densities resulted in large leakage currents, which in turn led to lower detectivities [60]. As mentioned above, while InSb and InAsSb do span a number of useful wavelengths, the precision gained in wavelength selectivity does come at a cost to the range of operating wavelengths. Additionally, the little response that InSb materials have in the LWIR regime is further reduced when operated at low temperatures, which is often necessary due to dark current considerations, due to reduced minority carrier lifetime in the n-type region of the material [4].

As InSb was a well-established technology as of 2000, numerous InSb photodetectors and FPAs had been built to this point. Most of the research performed in the decade was aimed at addressing specific concerns and problems that the technology faced. The two most common problems that this research addressed in the early part of the decade were increasing FPA performance and attempting to utilize different substrate materials in the devices. Universities typically focused on smaller FPAs with other performance improving features (e.g., an InAlSb current blocking layer in the active region of the detector) while companies, most notably Raytheon, focused on expanding pixel counts in FPAs beyond the megapixel range, out as far as 2K × 2K pixels. Whereas many single pixel devices were capable of operating at near room temperature, the FPAs were typically operated at liquid nitrogen (LN_2_) temperatures or lower to achieve the levels of performance desired [65–70].

Despite the difficulties growers faced when using substrates other than InSb, research continued on InSb IRPDs fabricated on these substrates. The most popular substrates (for reasons of cost, availability, and integration with other technologies) were silicon (*a* = 5.431), which has a lattice mismatch with InSb of 19%, and GaAs (*a* = 5.653), which has a lattice mismatch of 15%. Growth on these substrates was accomplished using a variety of means, most commonly MBE growth with the incorporation of buffer layers to reduce the strain at each interface. A number of devices were fabricated on both silicon and GaAs substrates, but these devices uniformly had higher dark current densities, lower responsivities, lower detectivities, and lower maximum operating temperatures than those fabricated on InSb substrates [60,61,63,71]. Other attempts were made at utilizing GaSb substrates, which does have the benefit of being much closer to a lattice match to InSb, with a lattice constant of 6.096 Å and a mismatch of 6%. The performance of these devices were generally more favorable than those on silicon or GaAs substrates, but operating temperatures were limited to 250 K and detectivities were still reduced [62].

As other technologies rose to prominence in the middle of the decade, research on InSb, particularly at universities, slowed. The Kuze group, one of the few groups that still performed research on these devices, focused on miniaturization of InSb sensors. These devices were constructed from a number of photodiodes connected in series with the aim of weak IR detection at room temperature. The final operational area of the devices was 600 × 600 μm, with final packaging 2.2 × 2.7 × 2.7 mm [72–75]. Other research looked into incorporating nitrogen into InSb. It was found that incorporating small amounts of nitrogen (<1%) led to improved D* at wavelengths as far out as 10 μm. While its incorporation did lead to a decrease in performance at shorter wavelengths, this device had among the highest performance in the LWIR of any InSb device [76]. Research into InAsSb devices has been fairly modest to this point. Most of the research has been focused on achieving the quality of growth required for devices, typically by limiting the mole fraction of As in the devices and including buffer layers. A handful of MWIR and LWIR IRPDs have been grown, achieving D* as high as 4.03 × 10^11^ cm Hz^1/2^/W at 170 K at a wavelength of 3.7 μm [76–78].

## 3. Quantum Well Infrared Photodetectors

**Figure 3.**
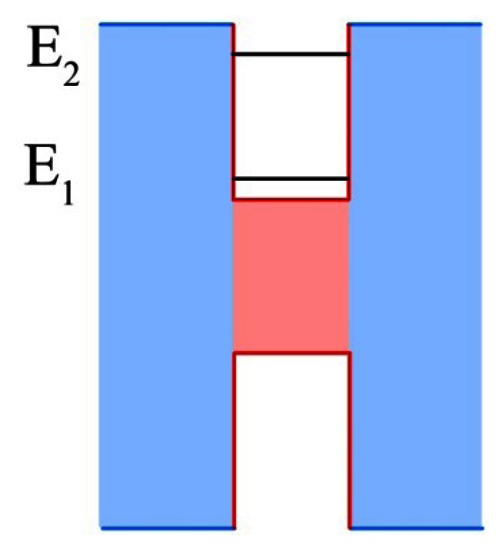
A band diagram of a quantum well in a QWIP. Carriers are excited by incident photons from the ground energy state of the well (*E_1_*) to the excited energy state of the well (*E_2_*).

Quantum well infrared photodetectors (QWIPs) operate on a much different principle than the bulk detectors that came before it. First demonstrated in 1987 by Levine *et al.* [79], QWIPs rely on quantum scale physical effects, whereas bulk detectors operate on larger scale effects. QWIPs in their most basic form are a periodic repetition of layers of two materials with dissimilar band gaps. The material with the lower energy band gap is commonly referred to as the well layer, while the higher energy band gap is referred to as the barrier layer. This structure approximates a series of quantum mechanical wells, with the well layer deposited thinly enough (generally on the order of nanometers to tens of nanometers) such that discretized energy states form within the well instead of the typical band of energy states. An example quantum well structure is provided in Figure 3. The energy states within the well will be characterized by Equation (5):

(5) $$E_{n}=\frac{{ћ}^{2}\pi^{2}n^{2}}{2m^{*}L_{w}^{2}}$$

where m* is the effective mass of the charge carrier, *L_w_* is the width of the well layer, and *n* is an integer identifying the energy level in the well [15]. The well layers are doped such that without illumination there will be carrier electrons present in the ground energy state. These carriers are then excited by incident photons to an energy state near the conduction band edge of the barrier material, where the applied voltage will sweep the carrier out of the well to the contacts. When the higher energy state is above the barrier conduction band edge (>10 meV), there is a higher escape probability from the well. This generally results in higher detectivities, and is referred to as a bound to continuum transition.

When the higher energy state is below the barrier conduction band edge (<10 meV) there is higher absorption efficiency; this is a bound to bound transition. Between these two values is a bound to quasibound transition and mixes a balance of the above traits. QWIP devices typically utilize an absorbing superlattice stack sandwiched between emitter and absorber layers, which aid in transferring carriers to and from the contacts and can be doped to assist with carrier separation. Due to the precise tolerances required for the layer thicknesses in the matrix of materials, these devices are typically grown using MBE, though other methods have been attempted with varying success [5,6,80].

QWIPs have a number of positive and negative performance characteristics that distinguish them from other IRPD technologies. In comparison to MCT devices, QWIPs are capable of lower dark currents, higher detectivities and higher NETDs. They make use of commonly used III-V material processing techniques, making fabrication of the devices easier than with MCTs. QWIPs also typically have greater radiation tolerances than narrower band gap materials like MCT and InSb. A case can be made that QWIPs have the greatest ease of material growth of multispectral imagers, as neither lattice mismatching (due to small layer thicknesses) or surface irregularities (due to a lack of nanostructures) are common hindrances for these devices [30,81–83].

QWIPs also face a number of challenges. The most distinct disadvantage arises from QWIPs' use of quantum confinement. Due to constraints defined by quantum mechanics, a QWIP can only absorb light that is incident upon it when there is quantum confinement in one of the perpendicular axes. This leads to a large problem: due to common growth techniques, most QWIPs are incapable of the absorption of normally incident light. In order to mitigate this problem, most QWIP devices use some form of a frontside scattering filter, which redirects normally incident light such that it can be absorbed [84].

It should also be noted that the maximum energy photon that a QWIP can absorb is limited by the energy difference between the conduction band edges of the materials. This means that for operation at shorter wavelengths, there must be a very large difference between the band edges. Finding materials that can satisfy this condition can be extremely difficult. For this reason, QWIPs have been used sparingly in the SWIR regime [85–87]. The scattering filters required for the absorption of normally incident light must be on a similar scale as the wavelength of the incident light. This complicates SWIR QWIPs by increasing the difficulty of fabrication as wavelength decreases. Those devices that attempt to operate at higher frequencies typically incorporate various nitride materials. There are also similar issues when the absorbed wavelength gets very long, into the VLWIR range [10,80].

QWIPs have significantly narrower absorption widths due to the discretized nature of the energy states transitions occur between. This can be advantageous if detection of a specific wavelength is required, but is a hindrance for wide band detection [81]. The narrow absorption bands also reduce photogenerated current, and hence the detectivity of QWIPs tends to be much lower than in bulk detectors. Lower detectivities in turn make QWIPs more sensitive to the magnitude of dark current density. As such, QWIPs have had significantly greater difficulty achieving high temperature operation [5,6,88,89]. Compounding the dark current issue is a short carrier lifetime in these devices, generally on the order of a handful of picoseconds. While a low carrier lifetime can have the added benefit of fast operating speeds, it also leads to increased sensitivity to dark current [90,91]. There are also a small number of issues that arise when considering QWIP FPAs. Due to the lower detectivities and responsivities of these devices, integration times tend to be longer when compared with bulk detectors (up to the order of milliseconds). This can make detection and identification of quickly moving objects more difficult [30].

Similar to bulk technologies, QWIPs had been the subject of research for some time as of 2000. Functional devices were common place and FPAs had recently begun to be developed. The vast majority of research conducted over the ensuing decade was performed by research labs at various universities. This section will focus first on single pixel QWIP designs, followed by various FPA designs, and finally with notes on a handful of technologies closely related to QWIPs, but which do not easily fit within the normal framework of QWIP devices.

One of the largest debates in the early portion of the 2000s was which material system held the most promise moving forward. Under consideration primarily were three material compositions (utilizing various mole fractions of the different ternary materials): (Al)GaAs/InGaAs, (In)GaAs/(Ga)InP, and AlGaAs/GaAs. Of primary interest during the earliest portion of the 2000s was the GaAs/InGaAs regime. It was hoped that this material system could help extend the functionality of QWIP devices out to the LWIR and VLWIR regimes (λ_c_ > 10 μm). Performance of these devices was generally not promising in these wavelength ranges, with required operating temperature often below LN_2_ levels (77 K). Even with these lower than normal operating temperatures, device performance was still lackluster, with responsivities regularly below 0.5 A/W and detectivities on the order of 10^9^ cmHz^1/2^/W. Performance of these devices was marginally improved with the incorporation of aluminum into the GaAs layer, with operating temperatures in ranges of LN_2_ cooling. Responsivities and detectivities were also higher after incorporation of aluminum, particularly at lower operating temperatures, peaking at around 1.96 A/W and 1.59 × 10^10^ cmHz^1/2^/W, respectively, by Lee *et al.* [92]. However, when quantum dot infrared photodetectors (QDIPs) began receiving attention as a viable alternative to QWIPs, particularly at these longer wavelengths, research interest in the GaAs/InGaAs material system petered out [93–98].

The next material system under serious consideration was InGaAs/(Ga)InP. This material regime also received the most attention at the beginning of the decade, with interest fading with the introduction of QDIPs. The material system was investigated across a wider range of wavelengths than the (Al)GaAs/InGaAs system, ranging from MWIR to VLWIR. InGaAs/(Ga)InP QWIPs met with similar difficulties in the LWIR and VLWIR regimes, with detectivities on the order of 1 × 10^9^ cmHz^1/2^/W around LN_2_ temperatures. These devices did exhibit stronger performance in the MWIR regime, feature operating temperatures over 100 K, responsivities as high as 2.2 A/W, and detectivities as high as 5 × 10^10^ cmHz^1/2^ [83,99–102]. There were also attempts made to adapt this material system for deposition on Si substrates, however performance of these devices is generally poorer than the normal performance of these devices, usually by an order of magnitude and sometimes more. This poor performance was attributed to the high density of defects and dislocations at the interface between the silicon substrate and the active QWIP stack [103,104].

By far the most commonly used material system over the decade was AlGaAs/GaAs. Whereas the other material systems often tried to extend operation out to the VLWIR regime, research with AlGaAs/GaAs typically focused on the MWIR and LWIR regimes. Dual band absorption was incorporated into a number of the structures, with performance of these devices often similar to single color devices. Performance of AlGaAs/GaAs devices outstripped that of the (Al)GaAs/InGaAs and (In)GaAs/(Ga)InP in the early portion on the decade, encouraging further research in the later portion of the decade [102,105–109]. There has been an effort to try and extend the operational wavelengths of AlGaAs/GaAs towards the VLWIR regime, but these efforts only met very limited success. Some reasonable responsivity values were achieved, but only at operating temperatures of 20 K. Performance at even LN_2_ temperature degrades rapidly [110–112]. Some have suggested the GaSb/AlGaSb material regime for use in the VLWIR wavelength range, but it remains sparsely investigated to this point [113].

Perhaps the most interesting results in the AlGaAs/GaAs material systems are those devices which incorporated some form of current blocking layer into the QWIP. A number of QWIP devices included such a layer, which consist of a significantly higher energy band gap intended to reduce the dark current that reaches the contacts. Such a layer can be implemented either within the active region or can be added between the complete material matrix and the other layers of the device. These devices generally exhibit improved dark current performance, but was not a topic of much interest. A number of the devices, while called QWIPs, can be seen as precursors to dots-in-a-well detectors (DWELLs), discussed later in this paper, particularly devices which incorporated the current blocking layer or layers into the material matrix. DWELLs have become increasingly interesting subjects of research in recent years, and seeing how they may have evolved from QWIPs is a fascinating occurrence [114–116].

In addition to single pixel QWIPs, a great amount of research was also performed on FPAs. Some of the research was focused on the InGaAs/InP material system. These devices, while showing some improvement over their single pixel predecessors (which can be attributed to improvement in MBE technology over the decade), still exhibited some of the same issues, namely dark current as much as an order of magnitude larger than AlGaAs/(In)GaAs QWIPs [117,118]. For this reason and the general superior performance over other material choices, most of the QWIPs FPAs researched utilized AlGaAs/(In)GaAs.

At the beginning of the decade, the most common size for these FPAs was 640 × 512 pixels. These devices were generally operated in the MWIR and LWIR regimes, and exhibited similar performance to single pixel devices. They were generally operated at LN_2_ temperatures with the strongest results produced by Gunapala *et al.*, with responsivities between 0.5–1 A/W and detectivities upwards of 2 × 10^11^ cmHz^1/2^/W. Spectral width, where reported, was generally between 10%–15% and NETDs between 30–50 mK [91,119–123]. As the decade progressed, efforts were made to increase the amount of pixels in the FPAs and their operating temperature. FPA sizes increased to over a megapixel in size, generally 1,024 × 1,024. While accomplishing this, operating temperatures were also raised, but the amount of improvement was fairly small, with the highest performance at just 105 K. NETD across these devices also improved through the decade, reaching below 25 mK for the highest performing devices [124–128].

### 3.1. Quantum Cascade Detectors

**Figure 4.**
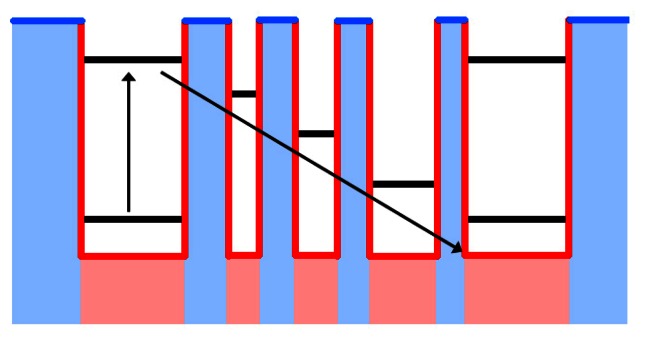
A band diagram of a quantum cascade detector. Incident light excites a carrier from the ground energy state of the absorbing well to an excited energy state. The carrier then tunnels through the barrier layers into the adjacent wells. This continues until the carrier reaches the ground state of the next period of the superlattice.

One interesting technology closely related to QWIPs is the quantum cascade detector (QCD). Like a QWIP, a QCD is a matrix of two alternating material layers: a well material and a barrier material. Whereas a period of a QWIP contains only two layers, one each of the well and barrier, each period of a QCD contains many layers. The first two layers are much like a period of a QWIP, with a long barrier layer followed by a collecting well layer. Much as in a QWIP, this collection well is doped such that electrons are present in the ground state of the well without illumination and upon illumination move to an excited energy state in the well, however unlike QWIPs this excited energy state is not near the conduction band edge of the barrier material. After these two layers are an additional series of thin layers, alternating between barrier and well materials. The thicknesses of these layers are chosen so as each successive well layer has an incrementally lower energy state than the excited energy state in the well. The carrier is able to tunnel through the adjacent barrier layer into the adjacent incrementally lower energy state. This continues through the rest of the period of the QCD until the carrier finally relaxes to the ground state of the absorbing well in the next period of the detector. The requirement for a number of excitations to produce a single carrier in the conduction band has a dramatic effect on the performance of the device. The band diagram of a QCD can be seen in Figure 4. This further reduces the detectivity and responsivity of the device, but with the benefit of also dramatically reducing the dark current generated [5,129].

## 4. Strained-Layer Superlattices

Since the first strained-layer superlattice (SLS) photodetector was grown from InGaAs/GaAs in 1984, superlattice (SL) and SLS structures have been of interest to the IRPD community [130]. Note: for brevity, in this publication, SL and SLS photodetectors will both be referred to as SLS, whether they are strained or not. SLS, while initially appearing to have a similar structure to QWIPs, actually operate using dramatically different physical principles. The misleading similarities result from a superlattice with extremely thin layer thicknesses (on the order of single nanometers) as an active absorbing layer. Additionally, while QWIPs generally utilize relatively thick barrier layers (on the order of tens of nanometers), in SLS the active layers have thicknesses on the same order of magnitude [131,132]. Like a QWIP, the most common structure for the superlattice is alternating layers of two different materials, but some more recent devices utilize more complex structures [30,133].

**Figure 5.**
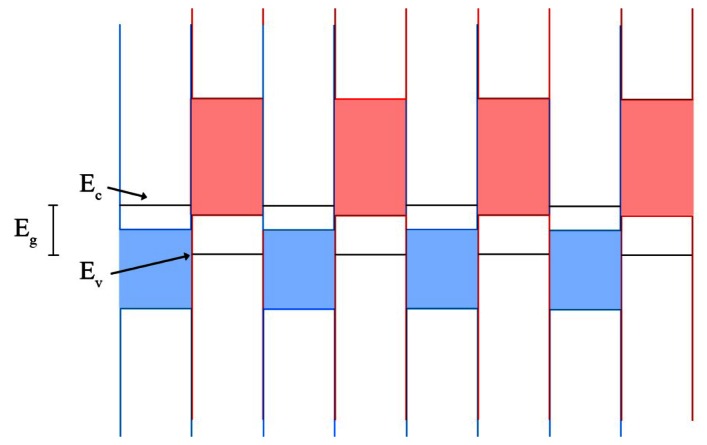
A band diagram of a InAs(blue)/GaSb(red) SLS. Carrier electrons are excited from the valence band of the GaSb layers to the conduction band of the InAs layers.

Similarities between the two technologies begin and end at the inclusion of a superlattice. QWIPs utilize a type-I band structure with explicit barrier and well materials, where the band edges of the smaller energy band gap are straddled by the band edges of the larger energy band gap. This structure allows QWIPs to utilize intraband transitions within the smaller band energy material in both the valence and conduction bands. SLS devices typically utilize a type-II band structure, where the band edges of the superlattice are staggered [134]. Due to this staggering of the band edges, each material acts as a well in one band (valence or conduction) and as a barrier for adjacent layers of the other material. An example SLS band structure can be seen in Figure 5. The thinness of the SLS layers allows for tunneling between wells in each band. Generally the barriers are thin enough that the available energy states in the wells of each band are able to effectively blur together despite being separated spatially in the different material layers of the superlattice, leading to what are commonly referred to as minibands or quasi-bands. It is the interband energy gap transitions between these minibands that determines the absorption wavelength of the device, rather than the band gap of either material (as in bulk detectors) or the spacing of energy levels within a single well (as in QWIPs and QDIPs). The devices are then biased to extract the charge carriers [133,135].

SLS have a number of performance strengths and weaknesses. SLS devices can be grown using many of the same techniques used for other III-V devices, but MBE growth is generally favored for its precise control of layer thicknesses and interfaces. Control of layer thickness, and particularly material interfaces, is vital for high performing devices. Poor quality interfaces can lead to increased band-to-band and defect-assisted tunneling currents. As SLS depends heavily on the spatial separation of charge for its performance, these tunneling currents are especially detrimental [136]. Materials chosen for use in SLS generally fall around the 6.1 Å lattice constant (InAs/GaSb/AlSb/*etc.*) with the most common superlattices comprised of InAs (electron capture) and GaSb (hole capture) layers [30,135]. Gallium-free versions also exist, using the same InAs layer for electron capture, but using an InAsSb for the hole capture. Use of InAsSb layers in place of GaSb would allow for longer wavelength absorption, but to this point research has been limited to characterization of superlattices comprised of these materials as opposed to fabrication of functioning photodetectors [137–139]. Due to the structure of SLS, dark currents tend to be fairly low for these devices. This in turn allows for higher operating temperatures compared to bulk detectors and QWIPs, particularly at longer absorption wavelengths. These detectors also allow normal incidence absorption, eschewing the need for frontside scattering filters. There have also been studies concluding higher values for the effective electron mass in these devices, which can also lead to reduced Auger recombination rates [133,140–142]. SLS devices also tend to be more tolerant to growth variances than MCT, particularly in terms of mole fraction composition [143].

Unfortunately, due to the overlap of the band structure of different materials in the devices, the effective band gap between the minibands tends to be fairly narrow. This tends to make it more difficult to use these devices for SWIR and shorter wavelength applications. It also makes SLS well suited for applications in the LWIR regime and longer wavelengths [133,144,145]. Due to high trap assisted tunneling rates in the active region of the device, there can be high amounts of non-thermal noise in these devices [146]. Additionally, the native oxides for many of the materials used in these devices are conductive. In FPAs, the sides of mesas can accumulate these oxides, which can enhance shunt current. Should adjacent pixels come into contact with each other due to oxide growth, those pixels will not perform as intended [133]. There can also be difficulty in achieving high quantum efficiency levels, as the thickness of the active regions can be small compared to other technologies, ranging from as small as hundreds of nanometers to as thick as a handful of microns [30,147].

In the early portion of the decade the majority of research in SLS was focused on InAs/GaSb devices grown on GaSb substrates. This material system had already been shown to function in previous work and various research groups sought to refine their utility. These devices were focused primarily on two miniband gaps: 150 meV (λ = 8.3 μm) and 80 meV (λ = 15.5 μm). These miniband gaps were chosen in part to fall squarely in the LWIR and VLWIR regimes, respectively, and also in part due to straightforward deposition ratios (42Å/42Å for the former, 20 ML (monolayers)/20 ML for the latter) [148,149]. Like QWIPs, some of these devices chose to incorporate current blocking layers into the superlattice. These devices showed promising performance in both regimes, capable of LN_2_ temperature operation even at this early stage. At these temperatures, the LWIR SLS with the best performance achieved detectivity on the order of 1 × 10^11^ cmHz^1/2^/W and responsivity of 1.2 A/W by Razeghi *et al.* Similarly, the best VLWIR device was capable of a D* and responsivity of 4.5 × 10^10^ and 4 A/W, respectively [148–153].

Moving forward, FPAs were created using SLS technologies. These FPAs began with small pixel counts, 256 × 256, and at this point were only utilized in the LWIR regime, but those produced were capable of similar performance metrics as the single pixel devices. As processing steps were refined, the imaging of these devices improved and an NETD of about 10 mK was achieved at LN_2_ temperatures [45,154,155]. Research on single pixel devices over this time frame focused on expanding the operation wavelengths SLS in both directions. SLS operational down to 5 μm were fabricated, maintaining the strong performance of previous tests with detectivities as high as 1.5 × 10^13^ cmHz^1/2^/W and responsivities remaining at 1 A/W by Krishna *et al.* [156–158]. It was also over this time frame that the first room temperature operation of an SLS IRPD, with a D* of 4.6 × 10^9^ cmHz^1/2^/W and responsivity of 2.2 A/W at 5.25 μm [144]. Also during this time frame the mechanics of this material system was researched, revealing information on its optical properties. It was discovered by Brown, Haugan, *et al.* that variation of the InAs layer thicknesses has a stronger effect on the band gap than variation of the GaSb layer thicknesses, and the dependence of the amount of interfacial strain on the number of periods in the superlattice [147,159,160].

Moving towards the end of the decade, research began to focus on production of larger and higher quality FPAs. The majority of these devices had cutoff wavelengths in the LWIR regime and were operated at LN_2_ temperatures. While none of the devices are capable of room temperature operation, a handful were capable of operation between 200 and 240 K. The expansion of FPA size, and perhaps the inclusion of other features such as current blocking layers into these devices, led to a small decrease in NETD, usually in the 20–30 mK range. These figures may seem low, however they are still more than sufficient for the purposes of an FPA [161–164]. By the end of the decade, a megapixel FPA had been fabricated, and its responsivities and detectivities remained similar to those found in previous FPAs. However, this FPA did suffer somewhat due to its size in the form of a higher NETD (over 50 mK) [165]. In addition to this FPA research, extension of previous work was performed, including optimization of growth conditions and further extending operational wavelengths. SLS were fabricated out to wavelengths of 4.3 μm. Other research focused on reducing the thickness of a single SLS period to 10 ML, which may enable this class of device to achieve noteworthy SWIR response, but this device resulted in lower detectivities at room temperature compared to other devices [136,145,162,164,166–168]. There were also some advancements in performance in recent years, with some single pixel devices with detectivities as high as 1.9 × 10^13^ cmHz^1/2^/W and responsivities as high as 1.33 A/W [169].

There was also some research performed on other material systems over this timeframe. The most common alternative to the InAs/GaSb system was InAs/InGaSb. Incorporation of indium into the GaSb layer allowed for increased flexibility in defining the energy level of the valence miniband. Performance of InAs/InGaSb devices was usually similar to devices utilizing InAs/GaSb structures. However, GaSb was usually the preferred due to more convenient material growth [143,170–172].

### 4.1. M/W Strained-Layer Superlattices

**Figure 6.**
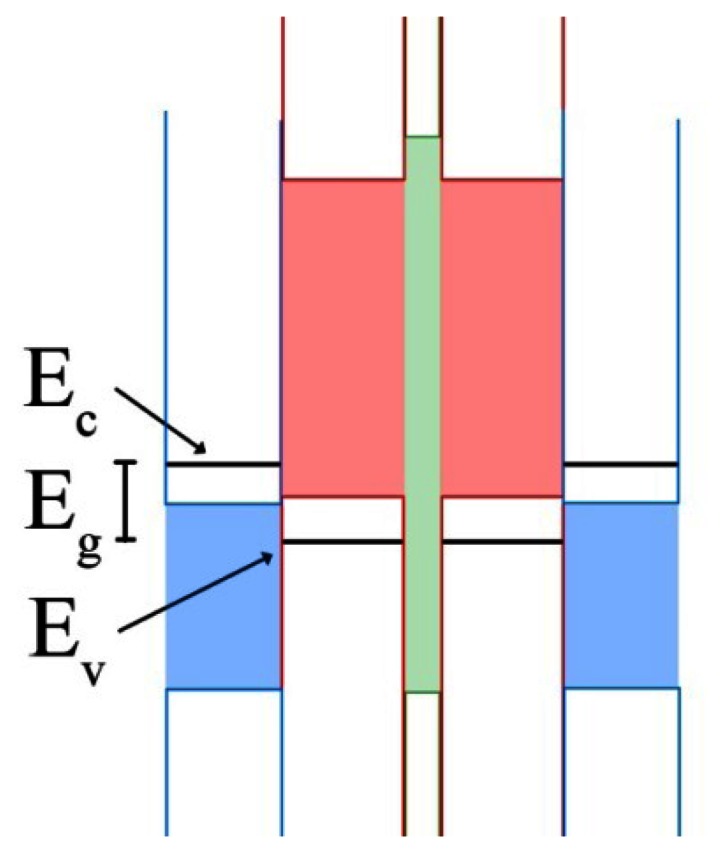
A band diagram of a InAs(blue)/GaSb(red)/AlSb(green) M-SLS. The inclusion of AlSb into the device gives this structure its eponymous band structure.

A pair of sub-technologies of SLS spawned due to the implementation of current blocking layers. Each of these technologies incorporates a different manner of a current blocking layer. The first, and arguably more complex, manner was first proposed in 2001, but not implemented until 2006 [173,174]. This technology incorporates a current blocking layer into one of the active superlattice layers, forming an ABCB superlattice as opposed to the traditional AB superlattice. This barrier layer typically has a significantly larger energy band gap than the surrounding material, leading to the distinctive band structure that gives these devices their name. An example band diagram can be seen in Figure 6.

The current blocking barrier, typically an aluminum bearing material, can be incorporated into either the InAs or GaSb layer, giving rise to the eponymous band structure in a period of the superlattice, resembling either a W or an M. The barrier placement in W- and M-superlattices is crucial in these devices. Inclusion of the barrier in the well shifts the two highest energy levels from the original well towards each other. When the barrier is precisely in the middle of the well the energy states become nearly degenerate. This in turn increases the number of energy states available at the desired energy level [164,175].

Even by the end of the decade, W- and M- superlattices were still a nascent technology. As such, much of the research performed is in the very early stages. Most single pixel devices have only been operated at LN_2_ temperatures. Only at the very end of the decade was an M-superlattice operated as high as 150 K. The results from that device, led by Razeghi *et al.*, with detectivities as high as 1.05 × 10^12^ cmHz^1/2^/W at 150 K in the MWIR regime, do show promise for this technology. Further, a 320 × 256 pixel FPA was constructed using this design, with imaging possible up to 170 K [131,174,176–178].

### 4.2. Unipolar/Monovalent Barrier Strained-Layer Superlattices

The other main technology incorporating current blocking layers in SLS are unipolar or monovalent barrier devices. In these devices, a single barrier layer is incorporated in the SLS structure immediately above or below (depending on whether the barrier is in the conduction or valence band) the active superlattice stack. The barrier material is chosen such that there is a relatively large barrier in the chosen band, but as little discontinuity as possible in the other band. This barrier allows the device to conduct photocurrent and diffusion current, but block both generation-recombination current and trap-assisted tunneling current [178–182]. Because of unavoidable variabilities in the growth process, most unipolar/monovalent SLS are operated at a slightly higher bias voltage to overcome the unintentional barrier than other devices, in case there is a discontinuity at the band edge between the barrier layer and the rest of the device [131].

**Figure 7.**
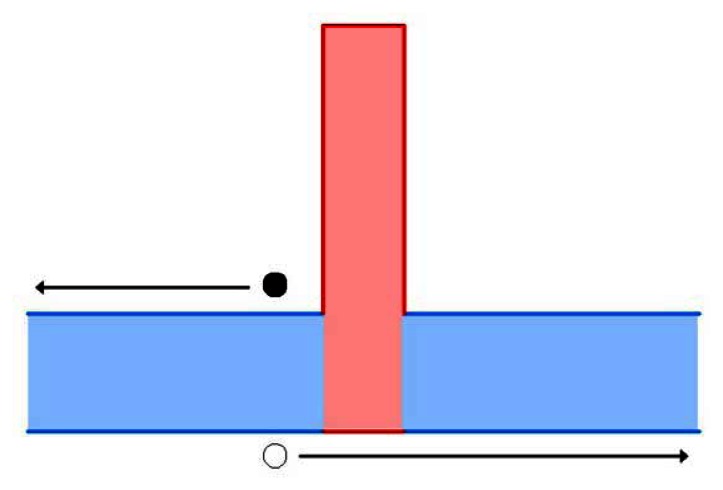
A band diagram of an nBn IRPD without applied bias. With the barrier in the middle, the left hand side acts as the photo-absorber. Absorbed photons create carrier pairs, which are unimpeded by the barrier. Thermally generated pairs on the right-hand side are blocked by the conduction band barrier, however.

**Figure 8.**
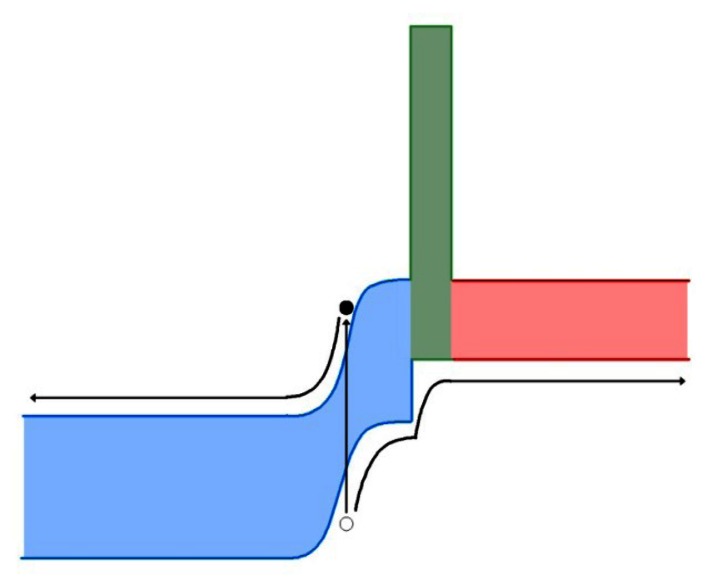
A band diagram of a pBn photodiode. A carrier electron is excited from the valence band of the n-type material and swept away from the barrier. The carrier hole is swept past the barrier layer, as it does not produce a potential barrier in the valence band of the p-n junction.

As with M- and W-superlattices, the first devices incorporating unipolar barriers did not arrive until the later part of the decade, 2006 [174]. With the explicit concern of limiting dark current, this class of devices quickly achieved room temperature operation. Monovalent barrier devices have been operated at a range of wavelengths from the MWIR to LWIR. One of the highest performing single pixel devices is capable of a D* of 1 × 10^9^ for a cutoff wavelength of 4.3 μm at room temperature. Barrier layers were investigated for both the conduction band (nBn structures) and the valence band (pBp structures), but nBn (Figure 7) were far more popular in the early stages of research [146,161,183–185]. A small FPA (320 × 256 pixels) was fabricated using nBn technology, having comparable performance to single pixel devices in the MWIR regime. Responsivities of 1.5 A/W, detectivities of 6.4 × 10^11^ cmHz^1/2^/W, and NETD of 24 mK were also achieved with nBn devices [163]. Recently, pBn have drawn interest for use in photodetectors, but very few devices have been successfully fabricated. To this point, pBn devices (Figure 8) have remained an exercise for theory and simulation, but promising results from early devices, included low dark current densities as low as 3 × 10^7^ A/cm^2^ at 150 K could lead to increased research with these devices [186–189].

## 5. Quantum Dot Infrared Photodetectors (QDIPs)

Quantum dots were initially posited in 1982 as a structure for use in quantum well lasers as a means to reduce or remove the temperature dependence from a laser's performance [190]. They remained a theoretical exercise for over ten years until 1993, when the first demonstrated growth of quantum dots was performed by MBE [191]. By 2000, research was well underway for quantum dot infrared photodetectors (QDIPs). In a QDIP, operation is very similar to that in a QWIP. A layer of dots, typically with diameters on the order of nanometers, is deposited on a well material, most commonly by MBE. These dots typically have narrower band gaps than the barrier material, creating a highly localized quantum well. A number of iterations are then deposited to improve the absorption of these devices [82,84,192,193–196]. Dots are typically fabricated using the Stranski-Krastanow growth mode, which requires a certain amount of lattice mismatch (between 1%–10%) between the dot material and the well material to ensure sufficient strain for islanding to occur. The thickness of the dot layer must also be precisely monitored, as excessive thickness (>2–3 ML for some systems) in the layer will change the surface morphology from quantum dots to a solid layer with large densities of dislocations [197]. Like QWIPs, QDIPs are doped such that there are electrons present in the conduction bands of the quantum dots. These electrons are then excited by incident photons to the conduction band of the well layers, where they can diffuse to the contacts of the device [9,15].

While much of the operation of QDIPs is reminiscent of that of QWIPs, there are a number of features which distinguish the former from the latter. The most notable difference is that, due to the quantum dot's confinement in all directions, QDIPs are capable of absorbing normally incident photons. The higher confinement also leads to lower dark currents, which can in turn lead to higher detectivities and operating temperatures [26,80,84,88,192,198,199]. QDIPs have longer carrier lifetimes than QWIPs, generally on the order of hundreds of picoseconds. However, the carrier lifetimes are shorter than in bulk detectors. This is believed to derive both from decreased amounts of phononic scattering in QDIPs and a phenomenon commonly referred to as phononic bottleneck. In this bottleneck, carriers fill the excited states of the dots. If these carriers are not extracted from the dots quickly, other carriers which would otherwise be excited to the higher energy level will not undergo that transition due to the lack of an available energy state [84,89,192,200]. Under ideal growth conditions, QDIPs also have higher selectivity and narrower spectral width absorption than QWIPs [8,192].

QDIPs do come with some disadvantages. While under ideal growth conditions QDIPs should have superior selectivity to QWIPs, in practice it is extremely difficult to have the kind of quantum dot size uniformity required for high levels of performance. Due to uniformity variance in the quantum dots, oftentimes QDIPs have wider spectral absorption than QWIPs [84,193]. For similar reasons, tuning QDIPs to a specific wavelength with great accuracy can be extremely difficult [15,201]. The dot layers in a QDIP are by their nature very uneven, leading to issues with strain and dislocations with an increasing number of absorption layers. As such, QDIPs often exhibit fewer absorbing layer iterations than QWIPs, leading to lower absorbances. To combat this effect, some QDIPs have strain reducing layers incorporated into their design. While this raises the complexity of the fabrication, it can lead to higher device performance. Quantum efficiencies (QEs) for QDIPs also tend to be much lower than QWIPs. This low QE is due to the relative paucity of energy states in QDIPs (a few monolayers of scattered dots in QDIPs *vs.* several nanometers of a solid layer in QWIPs), lower absorption of incident light, and lower capture probabilities by individual dots due to more sharply defined energy states than in QWIPs [84,193,202].

The vast majority of research related to QDIPs over the course of the decade was related to four main material regimes: InAs dots in GaAs barriers, InGaAs dots in GaAs barriers, InGaAs dots in InGaP barriers, and InAs dots in InGaAs barriers. Of these, InAs dots in GaAs barriers was the most common device in the early part of the decade. QDIPs using InAs dots in GaAs barriers typically operate in the SWIR and MWIR regimes, with a handful out as far as 7 μm. In the devices of the early part of the decade there was significant difficulty in growing numerous iterations of absorbing layers due to a buildup of strain, exacerbated by the irregular surface morphology of the dots. As such, detectors rarely exceed ten layers. As might be expected, the low number of absorbing layers led to fairly low detectivities, as high as 2.94 × 10^9^ cmHz^1/2^/W at LN_2_ temperatures by Madukar *et al.* [15,203–205]. It was in the early part of the decade that some of the earliest InAs/GaAs FPAs were fabricated. While the imaging of these devices was not superlative, as they were intended as proofs of concept more than final designs, they do produce recognizable images. Due to the low dark current densities present, even the initial devices were capable of operating up to 150 K in the MWIR regime [88,206]. As the decade moved forward, progress addressed a number of QDIP issues. Most notably was the number of quantum dot layers which could be deposited without dislocations. Whereas in the early part of the decade, layer numbers were limited to ten or less, by 2005 layer numbers InAs/GaAs QDIPs were in 50–70 range. Detectivities rose some with this increase, to a high of 1.2 × 10^11^ cmHz^1/2^/W [207]. It should be noted that even in these later devices, responsivities were still low, with a high value of 0.3 A/W [193,208,209]. Advances were also made with FPAs, with a more refined device fabricated in the middle of the decade. A 256 × 256 pixel FPA with similar performance metrics was fabricated with significantly improved imaging capabilities over previous devices [210]. However, following the middle part of the decade research on InAs/GaAs QDIPs slowed, replaced by research into the other material systems.

A different pair of the material systems had been under limited research over the past decade. The first of these systems was InGaAs dots on GaAs barriers. These materials offer increased design flexibility, as dot composition can be varied along with dot size. This system was researched more towards the later portion of the decade, as interest in the InAs/GaAs system waned. Operation temperatures InGaAs/GaAs QDIPs were comparable to InAs/GaAs devices. Early devices also had a number of the same issues as InAs/GaAs, including a low number of quantum dot layers and low detectivities. By the end of the decade, FPAs using InGaAs/GaAs were outperforming InAs/GaAs FPAs, capable of detectivities of 1.01 × 10^11^ cmHz^1/2^/W and responsivities of 2.1 A/W by Jagadish *et al.* [15,211–213]. The other lesser used material system involved InGaAs dots in InGaP barriers. However, these devices generally had poor performance, including low quantum efficiencies and fill factors. These low metrics have been attributed to the lower lattice constant mismatch (there is a mismatch of about 3.2% between InGaAs and InGaP, whereas InAs/GaAs devices have a mismatch of about 7%) [214–217].

The last common material system that has been investigated was InAs dots in InGaAs barriers. As with other material systems for QDIPs, research for this system did not begin getting traction until the middle portion of decade when researchers were looking to improve performance from InAs/GaAs devices. These InAs/InGaAs devices functioned in the MWIR and LWIR regimes and operated at temperatures as high as 190 K. QDIPs of this system which operated at longer wavelengths did tend to suffer in terms of D*, only reaching as high as 4.6 × 10^9^ cmHz^1/2^/W at LN_2_ temperatures. However, when operated in the MWIR, these structures achieved comparable performance metrics to other material systems. Some of these devices, as well as some of the InGaAs/GaAs and InGaAs/InGaP devices, were grown by MOCVD, as opposed to MBE, with little hindrance on performance. MOCVD growth has an advantage to potentially make larger scale fabrication of QDIP devices much easier [218–221]. A 350 × 256 pixel FPA QDIP was fabricated near at the end of the decade. While operation of this device was constrained to LN_2_ temperatures, D* at that temperature was as high 1 × 10^10^ cmHz^1/2^/W by Lai *et al.*, which was among the highest for a QDIP FPA of any type [222].

A material system that may be interesting for future QDIP devices is GaAs dots on GaSb substrates. While this material system has not been used in a functioning IRPD, it has been used in other optical devices effectively, including lasers, solar cells, and LEDs, most notably by the Huffaker group [223–227]. For these devices, both Stranski-Krastanov and, more commonly due to the size of the lattice mismatch, interfacial misfit growth modes have been utilized. Most QD devices utilizing this material system have shown peak photoluminescence in the 1–2 μm range, which could make it suitable for NIR applications [228,229].

Another interesting subset of QDIPs utilized a pair of resonant tunneling layers incorporated into the periodic absorbing stack. In these structures, a quantum dot is deposited on a barrier material. This is followed by deposition of a very thin layer of a higher energy band gap material, a thin layer of the original barrier material, and a second thin layer of the higher energy band gap material. This creates two energy wells in each layer of the absorbing stack of the device, albeit at a much higher energy level than the quantum dot well. The intention is that by tuning the energy level of this second energy well, the well will transmit photocurrent excited from the initial well. However, the well won't transmit a large fraction of the dark current. These devices, while reducing dark current and maintaining respectable detectivities, encountered great difficulty in terms of overall responsivity, not capable of achieving a responsivity of 0.1 A/W at LN_2_ temperatures. This low responsivity and the additional complexity of the structure has limited the appeal of these devices [230,231].

Another interesting subset of QDIP devices is that which incorporate periodic current blocking layers. The current blocking layers (usually composed of either AlGaAs or InGaAs) were mainly incorporated at the early portion of the decade and were typically incorporated as capping layers on the quantum dots. The unintended consequence, at least in early devices, was the effect they had on the energy states in the quantum dots, particularly with InGaAs layers. These current blocking layers, if thick enough, could change the level of the energy states in the quantum dot or create entirely new energy levels in the barrier or well materials. While not having the explicit aim or the ultimate polish of later devices, it is easy to see these devices as precursor to quantum dot-in-a-well photodetectors (DWELLs). These early DWELL devices were plagued by many of the same issues as resonant tunneling barrier QDIPs, including low responsivities and low counts of quantum dot layers. However, they were able also to achieve detectivities up to 1.1 × 10^12^ cmHz^1/2^/W. Ultimately, a 320 × 256 pixel FPA was fabricated using these techniques, which was capable of imaging at 120 K, albeit with an NETD of 344 mK [213,220,232–235].

## 6. Quantum Dots-in-a-Well Photodetectors (DWELL-IPs)

Utilization of structures called DWELLs in IRPDs began in 2002, though the structure was previously used in other applications, such as semiconductor lasers [33]. While 2002 is the earliest direct reference to a DWELL structure, other structures did utilize various capping layers in QDIPs. These structures unintentionally made the introduction of DWELLs to IRPDs harder to pin down. Over the next couple years there was a gradual delineation between the DWELL structures and QDIPs. Once that delineation had been made, research on devices specifically labeled DWELLs picked up.

The physical structure of a DWELL is much akin to a combination of a QWIP and a QDIP. A layer of the absorbing stack begins with deposition of a wide band gap material, the barrier material. This deposition is followed by growth of a narrower band gap material, the well material, much as in a QWIP. Growth is then followed with deposition of a layer of quantum dots with a band gap that is narrower still, known as the dot material. At this point, some devices will add a capping layer, sometimes composed of the well material [236]. Because these devices typically require precise tolerances, MBE deposition is typically the method of growth chosen for DWELLs. The absorbing layers will then be repeated a number of times to improve the absorption of incident light [8].

**Figure 9.**
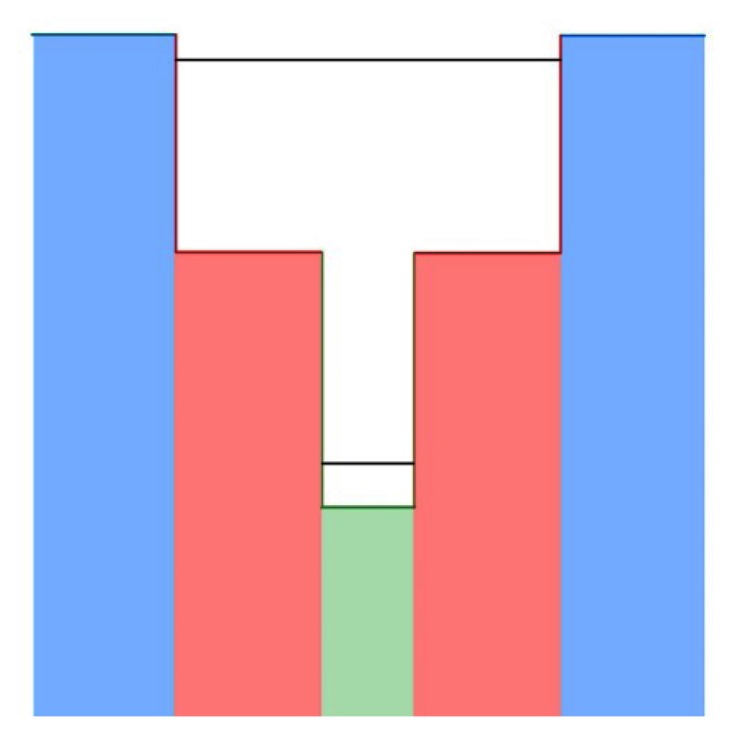
A band diagram of a DWELL structure, with a barrier layer (blue), well layer (red), and dot layer (green).

The structure of the DWELL is designed to address some of the weaknesses of both QWIPs and QDIPs. One of the most notable flaws of the QWIP is the inability to absorb normally incident light. The inclusion of a quantum dot layer allows DWELLs to utilize light at normal incidence. QDIPs can have difficulty in precisely controlling the energy levels in the quantum dots, as those energy levels depend heavily on dot size. The inclusion of a well layer in addition to the quantum dot layer adds significantly greater control over the energy level spacing. The well and dot layers, being adjacent, form what amounts to a combined energy well. For DWELLs which utilize two energy states in the well, the ground state of the well occurs in the dot, with the absorbing layer doped to the point such that electrons populate the lowest energy state in the dot at rest (Figure 9). The next energy level is designed to be located in the quantum well layer. The location of this energy level can be adjusted by varying the thickness of the well layer. This can allow for easier wavelength selectivity, as that selectivity can be determined by varying layer thicknesses instead of quantum dot diameters. The amount of flexibility in the design, in terms of dot size, material choices, well thickness, *etc.*, has also led to alternative methods of multispectral imaging. While multispectral imaging is available in a number of different technologies, it is generally implemented by stacking detectors for two different wavelengths monolithically. In DWELLs, there is enough flexibility such that two or three energy transitions can exist within the well. Each transition then corresponds to a different absorption wavelength [8,33,237].

While DWELLs do address a number of the weaknesses present in QWIPs and QDIPs, the technology still has some of the faults present in other devices. One of the major difficulties DWELLs have in common with QDIPs is a low number of absorbing layers which can be grown on a single device. As with QDIPs, use of the quantum dots can lead to strain build-up. As more layers are deposited, the strain accrues, and if too many layers are deposited defects can form. Accumulations of defects limit the amount of absorbing layers that can be deposited in a DWELL, which can in turn lead to low absorbances and quantum efficiencies. Much like in QDIPs, this buildup of strain can be mitigated with the inclusion of strain reducing layers at the expense of device complexity [238].

Many of the earliest devices which utilized DWELLs existed either as proofs of concept or confirmation of theoretical predictions. Characteristics, such as the effect of variation of quantum dot size on absorption wavelength (smaller dots absorbed shorter wavelengths), the effect of variation of well layer thickness on absorption wavelength (thicker layers led to longer absorbed wavelengths), and the effect of variation of well layer mole composition (narrower band gaps led longer absorption wavelengths), were all investigated. Other growth characteristics were also characterized in this time frame. The effect of overall deposition speed on device performance was examined, with slower growth speeds associated with higher photoluminescent response. The effect of growth speed proved to be most important with the quantum dot layer, as the lower growth speed allows for greater uniformity of quantum dot size, which in turn leads to better performance at the desired absorption wavelength. Similarly, higher quantum dot densities were also associated with stronger response, but were much easier to achieve with slower deposition speeds [8,82,239,240].

Most of the early DWELL devices had a similar structure. Operating temperatures were typically around that of LN_2_, though some early designs did achieve operation near 100 K. These devices utilized a structure of GaAs barriers, some composition of InGaAs for the wells, and InAs quantum dots. The barriers were typically tens of nanometers thick, had well layers under ten nanometers, and contained quantum dots 1–3 ML thick. The variations in layer thicknesses were enough to dramatically alter devices absorption characteristics. Early DWELL-IPs spanned a wide range of absorption wavelengths, from the MWIR to VLWIR. A number of early devices also exhibited multispectral absorption from multiple available energy transitions within individual wells [33,201,241]. By the middle portion of the decade, a small 320 × 256 pixel FPA was produced, though for this early device responsivity and quantum efficiency was very low [8]. Dark current levels in these early devices were generally lower than those in QWIPs, but still higher than those in QDIPs. Responsivities varied significantly depending on the absorption wavelength, but responsivities in the MWIR regime as high as 3.58 A/W were achieved by Krishna *et al.* At longer wavelengths, this value decreased dramatically, with some of the VLWIR regime devices having responsivities as low as 0.01–0.02 A/W by Krishna *et al.* [33].

Moving through the decade, research continued on DWELLs at universities nationwide. Operating wavelengths were extended out further into the VLWIR regime. Often, long wavelength detection occurred as the secondary or tertiary absorption wavelength in the DWELL. As long as the devices were operated at low temperatures (sometimes as low as 5 K) detection of VLWIR radiation was still possible [242,243]. A number of groups continued characterizing the effect of various growth conditions and deposition characteristics on DWELL performance. Variation in the doping density for DWELLs was investigated by Attaluri, Krishna, *et al*, with higher doping densities leading to both larger spectral responses and large dark current densities. It was also theorized that doping DWELLs to the point that there was an average of two electrons per quantum dot (opposed to the lower numbers normally used) could lead to a decrease in photocurrent due to overfilling energy states in the well [89]. Perera, Krishna *et al.* investigated the effect of annealing on DWELL performance. Annealing was found to increase the responsivity of the device, but also increased the dark current and shifted the absorption peak by up to 3.5 μm in wavelength. The change in performance is attributed to the effect of annealing on the quantum dots. It is believed that annealing increased material diffusion between the dot and the capping layer and, therefore, reduces the confinement of the quantum dots in devices. Despite these advances, the operating temperatures of DWELL devices remained fairly low, rarely operating significantly above LN_2_ temperatures and even then barely above them [244–246]. Double well DWELLs, where one period of the DWELL absorbing incorporates a barrier layer, two well layers, and a dot layer, were also developed over this time period, led by the Krishna group. In these structures, the dot layer is generally InAs, the first well is a thin layer of InGaAs to aid in the formation of the quantum dots, the second well layer is GaAs, and the barrier layer is AlGaAs. Less strain develops in a DWELL using this structure, allowing for more iterations of the absorbing layer to be deposited in a single device [247–249]. Using this structure, devices with up to 80 periods have been fabricated. These devices operated in both the MWIR and LWIR regimes and performed similarly to normal DWELL devices with a handful of minor distinctions. These devices tended to have marginally lower responsivities (around half that of single well DWELLs), while have improved NETDs in FPAs, reaching as low as 105 mK [250]. These devices also were capable of D* as high as 3 × 10^10^ cmHz^1/2^/W at LN_2_ temperatures by Krishna *et al.* [251–258].

Other growth methods besides MBE were attempted for DWELLs, with the intention of easing fabrication of the devices. These alternative methods still required fine control of deposition speeds, to accommodate quantum dot growth, and the ability to deposit a variety of materials without breaking vacuum. The first alternative method was MOCVD. Working DWELL devices were fabricated using MOCVD, though it should be noted that the devices produced had notably lower responsivities and detectivities at LN_2_ temperatures. These devices performed in the range of 0.05–15 A/W and 1–6 × 10^9^ cmHz^1/2^/W, respectively. Additionally, a small change to the typical DWELL structure was made in these devices. Instead of using InGaAs for the well material and GaAs for the barrier material, these devices used GaAs for the well material and AlGaAs for the barrier material. The effect in terms of the band diagram of the DWELL structure is effectively the same, with only slightly different differences in the band edge locations. The use of these two materials does have one particular advantage. Compositions of AlGaAs are closely lattice-matched to GaAs, whereas InGaAs (particularly compositions with high indium contents) may not necessarily have the same trait. This trait is of particular significance when changing to a lower quality growth method, such as from MBE to MOCVD, as the larger mismatch can lead to high densities of defects [259,260].

A number of DWELL designs utilized similar resonant structures that have been mentioned previously for other IRPD technologies. These designs implemented a resonant tunneling barrier, typically located in the well layer of the DWELL. These layers are typically a pair of wide band gap layers of AlGaAs with a layer of InGaAs in between. Notably, the AlGaAs layer has a wider band gap than the barrier of the normal DWELL structure and the InGaAs layer has a narrower band gap than the band gap of the well material. As in the previous examples, these layers are included to attempt to reduce dark current and improve detectivity. The DWELL structures utilizing the resonant tunneling structures did exhibit strong D*, as high as 2.9 × 10^10^ cmHz^1/2^/W in the LWIR regime at LN_2_ temperatures. However, they also suffered in terms of device responsivity [261–263]. There was additional progress made in the development of DWELL FPAs during this time frame. FPAs as large as 640 × 512 pixels were fabricated for MWIR applications at LN_2_ temperatures. This device has a D* as high as 1 × 10^10^ cmHz^1/2^/W and an NETD of 40 mK [264].

## 7. Discussion

Tables 1, 2, 3, 4, 5 and 6 list the highest performing devices of different IRPD technologies across different wavelength regimes. In these Tables, a number of the most common IRPD performance metrics are examined. Results which stray too far from these standard conditions and metrics, or utilize wholly different metrics for characterization, are not included in these Tables. Detectivity (D*), both at LN_2_ and at the highest temperature with successful results are reported. Similar results are reported for responsivity (*R*). The highest temperature at which a device has been successfully operated is also reported. The lowest dark current density (*J_dark_*) at LN_2_ temperatures is included in the table. Finally, to compare FPA performance, a comparison of noise equivalent temperature difference (*NETD*) is also included.

## 8. Conclusions and Future Prospects

The progress that the field of infrared photodetection has made in recent history is tremendous. What started as primarily academic research has transformed into a budding industry, including a number of new technologies in the pipeline. IRPDs will see increasing use in a number of fields in the present and near future, ranging from military target detection to environmental sensing. With the number of applications for these technologies rapidly increasing, there is a need to determine which technology is best suited to each application.

The number of different IRPD technologies has led to segmentation of which is currently the most viable depending on the application. Bulk detectors have achieved the most commercial penetration due to ease of large scale fabrication, long track record of growth, flexible absorption wavelengths (which can span from SWIR to LWIR), and high detectivity at cryogenic temperatures, but there has been difficulty in raising the operating temperatures of these devices while maintaining performance. QWIPs and QDIPs have also drawn significant attention due to the former's capability to perform in the VLWIR regime and the latter's high temperature performance, currently the best among current devices. DWELL-IPs and SLS devices have shown noteworthy promise in terms of performance, range of absorption range, and operating temperature. It may well be that these devices could soon overtake QWIPs and QDIPs in terms of performance.

Moving forward, progress in the field of IRPDs may come from dramatically different sources than research to date. In recent years, there has been a shift in the source of funding for some IRPD technologies. The increase in research conducted by private businesses in these technologies has led to a change in project selection from many funding agencies, viewing research in these fields increasingly as industrial development as opposed to academic research. That academic research that is funded has a greater focus on producing front-end devices, which may eventually lead to commercial ventures, as opposed to investigating novel applications or phenomenon in the established technology. This has primarily occurred with bulk detectors so far, primarily with MCT and InSb, but as other technologies become more prevalent this trend will likely spread across the field.

## Figures and Tables

**Table 1.** Highest reported performance metrics for HgCdTe IRPDs across different wavelength regimes.

**Metric**	***SWIR***	***MWIR***	***LWIR***	***VLWIR***
$D_{LN_{2}}^{*}\;[{\text{cmHz}}^{1/2}/W]$	1 × 10^14^ [265]	2 × 10^13^ [265]	2 × 10^13^ [265]	X
$D_{T_{\text{high}}}^{*}[{\text{cmHz}}^{1/2}/W](T_{\text{high}}[K])$	1 × 10^12^ (300) [265]	7 × 10^10^ (210) [25]	1 × 10^7^ (300) [52]	X
*R_LN_2__* [(*A*/*W*)]	X	X	1 [266]	X
*R_T_high__*(*T_high_*) [*A*/*W*]	1 [24] (300)	X	X	X
*T_max_* [*K*]	300	210 [25]	300	100 [35]
*J_dark_* [*A*/*cm*^2^]	X	X	2.5 × 10^−4^ [36]	2.7 × 10^−7^ [41]
*NETD* [*mK*]	20 [24]	10 [267]	9 [55]	22 [41]

**Table 2.** Highest reported performance metrics for InSb IRPDs across different wavelength regimes.

**Metric**	***SWIR***	***MWIR***	*LWIR*	***VLWIR***
$D_{LN_{2}}^{*}[{\text{cmHz}}^{1/2}/W]$	X	1 × 10^10^ [63]	7.6 × 10^8^ [64]	X
$D_{T_{\text{high}}}^{*}[{\text{cmHz}}^{1/2}/W](T_{\text{high}}[K])$	X	2.8 × 10^8^ (300) [73]	X	X
*R_Ln_2__* [(*A*/*W*)]	X	1.4 [60]	X	X
*R_T_high__*(*T_high_*)[*A*/*W*]	X	X	X	X
*T_max_* [*K*]	X	300 [73]	300	X
*J_dark_* [*A*/*cm*^2^]	X	X	X	X
*NETD* [*mK*]	X	2.2 [72]	X	X

**Table 3.** Highest reported performance metrics for QWIPs across different wavelength regimes.

**Metric**	***SWIR***	***MWIR***	***LWIR***	***VLWIR***
$D_{LN_{2}}^{*}[{\text{cmHz}}^{1/2}/W]$	X	2 × 10^11^ [116]	2 × 10^11^ [91]	5 × 10^9^ [121]
$D_{T_{\text{high}}}^{*}[{\text{cmHz}}^{1/2}/W]$	X	1 × 10**^11^** (210) [107]	3.2 × 10**^10^** (110) [99]	5 × 10^9^ (70) [121]
*R_LN_2__* [(*A*/*W*)]	X	1 [268]	1.4 [118]	0.38 [119]
*R_T_high__*(*T_high_*)[*A*/*W*]	X	0.7 (290) [107]	0.3 (120) [92]	0.25 (70) [121]
*T_max_* [*K*]	X	290 [107]	120	70 [121]
*J_dark_* [*A*/*cm*^2^]	X	1 × 10^−7^ [116]	1 × 10^−9^ [99]	1.7 × 10^−3^ [109]
*NETD* [*mK*]	X	14 [123]	19 [118]	48 [122]

**Table 4.** Highest reported performance metrics for QDIPs across different wavelength regimes.

**Metric**	***SWIR***	***MWIR***	***LWIR***	***VLWIR***
$D_{LN_{2}}^{*}[{\text{cmHz}}^{1/2}/W]$	1 × 10^9^ [203]	1 × 10^12^ [235]	1 × 10^11^ [213]	X
$D_{T_{\text{high}}}^{*}[{\text{cmHz}}^{1/2}/W]$	1 × 10^9^ (77) [203]	2.4 × 10^8^ (250) [269]	1.2 × 10**^11^** (77) [207]	2 × 10^8^ (40) [15]
*R_LN_2__* [(*A*/*W*)]	1 [203]	2.5 [270]	5.3 [232]	X
*R_T_high__*(*T_high_*)[*A*/*W*]	1 (77) [203]	0.012 (300) [270]	5.3 (77)[232]	4 (40) [15]
*T_max_* [*K*]	77 [203]	300 [270]	77	40 [15]
*J_dark_* [*A*/*cm*^2^]	X	1 × 10^−13^ [193]	3 × 10^−8^ [232]	X
*NETD* [*mK*]	X	87 [214]	X	X

**Table 5.** Highest reported performance metrics for DWELL IRPDs across different wavelength regimes.

**Metric**	***SWIR***	***MWIR***	***LWIR***	***VLWIR***
$D_{LN_{2}}^{*}[{\text{cmHz}}^{1/2}/W]$	X	1 × 10^10^ [271]	3 × 10^10^ [256]	X
$D_{T_{\text{high}}}^{*}[{\text{cmHz}}^{1/2}/W]$	X	1 × 10^8^ (250) [272]	1 × 10^8^ (200) [256]	X
*R_LN_2__* [(*A*/*W*)]	X	0.12 [259]	3.58 [33]	X
*R_T_high__*(*T_high_*)[*A*/*W*]	X	0.12 (77) [259]	0.2 (110) [244]	0.025 (4.6) [8]
*T_max_* [*K*]	X	77	110 [244]	4.6 [8]
*J_dark_* [*A*/*cm*^2^]	X	1 × 10^−5^ [271]	5 × 10^5^ [261]	X
*NETD* [*mK*]	X	X	40 [264]	X

**Table 6.** Highest reported performance metrics for SLS IRPDs across different wavelength regimes.

**Metric**	***SWIR***	***MWIR***	***LWIR***	***VLWIR***
$D_{LN_{2}}^{*}[{\text{cmHz}}^{1/2}/W]$	X	1.9 × 10^13^ [169]	1 × 10^12^ [155]	4.5 × 10^10^ [150]
$D_{T_{\text{high}}}^{*}[{\text{cmHz}}^{1/2}/W](T_{\text{high}}[K])$	1 × 10^8^ (300) [166]	4.6 × 10^9^ (300) [144]	X	4.5 × 10**^10^** (80) [150]
*R_LN_2__* [(*A*/*W*)]	X	1.33 [169]	3.2 [162]	4 [150]
*R_T_high__*(*T_high_*)[*A*/*W*]	X	2.2 (300) [144]	X	4(80) [150]
*T_max_* [*K*]	300 [166]	300	180 [162]	80 [150]
*J_dark_* [*A*/*cm*^2^]	X	1 × 10^−7^ [163]	1 × 10^−3^ [171]	3.3 × 10^−3^ [184]
*NETD* [*mK*]	X	10 [178]	270 [162]	X
